# Assaying Chromatin Accessibility Using ATAC-Seq in Invertebrate Chordate Embryos

**DOI:** 10.3389/fcell.2019.00372

**Published:** 2020-01-24

**Authors:** Marta Silvia Magri, Sandra Jiménez-Gancedo, Stephanie Bertrand, Alicia Madgwick, Hector Escrivà, Patrick Lemaire, José Luis Gómez-Skarmeta

**Affiliations:** ^1^Centro Andaluz de Biología del Desarrollo, CSIC-Universidad Pablo de Olavide-Junta de Andalucía, Seville, Spain; ^2^Sorbonne Université, CNRS, UMR 7232, Biologie Intégrative des Organismes Marins, Observatoire Océanologique, Banyuls-sur-Mer, France; ^3^Centre de Recherche en Biologie Cellulaire de Montpellier, CNRS, UMR 5237, Université de Montpellier, Montpellier, France

**Keywords:** ATAC-seq, *cis*-regulatory elements, invertebrate chordates, amphioxus, tunicates, development, evolution

## Abstract

*Cis*-regulatory elements (CREs) are non-coding DNA regions involved in the spatio-temporal regulation of gene expression. Gene regulatory changes drive animal development and play major roles during evolution of animal body plans. Therefore, we believe that determining CREs at different developmental stages and across animal lineages is critical to understand how evolution operates through development. The Assay for Transposase-Accessible Chromatin followed by high-throughput sequencing (ATAC-seq) is a powerful technique for the study of CREs that takes advantage of Tn5 transposase activity. Starting from fewer than 10^5^ cells, in a 1-day procedure, it is possible to detect, at a genome-wide level, CREs located in open chromatin regions with high resolution. Here, we describe a detailed step-by-step ATAC-seq protocol for invertebrate chordate marine embryos. We have successfully applied this technique to amphioxus and two species of tunicate embryos. We also show an easy workflow to analyze data generated with this technique. Moreover, we point out that this method and our bioinformatic pipeline are efficient to detect CREs associated with Wnt signaling pathway by simply using embryos treated with a drug that perturbs this pathway. This approach can be extended to other signaling pathways and also to embryo mutants for critical genes. Our results therefore demonstrate the power of ATAC-seq for the identification of CREs that play essential functions during animal development in a wide range of invertebrate or vertebrate animals.

## Introduction

During embryonic development, cellular cross-talks must be tightly coordinated to allow the proper formation of the different tissues in the developing organism. These cross-talks are mediated by a limited number of pleiotropic signaling pathways that precisely control gene expression. Developmental genes are among the main targets of these signaling pathways. Accordingly, the expression of these genes is precisely regulated in space and time by the combined action of different pathways. However, how developmental genes integrate these multiple inputs is largely unexplored.

*Cis*-regulatory elements (CREs) are non-coding DNA regions involved in the regulation of gene expression. These CREs, mainly constituted of enhancers and promoters, contain DNA binding sites for transcription factors (TFs). TFs binding at CREs facilitate the recruitment of the RNA polymerase II machinery at the promoter of target genes, triggering their expression. Signaling pathways operate through different families of TFs. Therefore, identification of the CREs bound by these TFs that respond to signaling pathways would help to unravel how developmental genes integrate these signals (Spitz and Furlong, 2012; Long et al., 2016).

**FIGURE 1:**
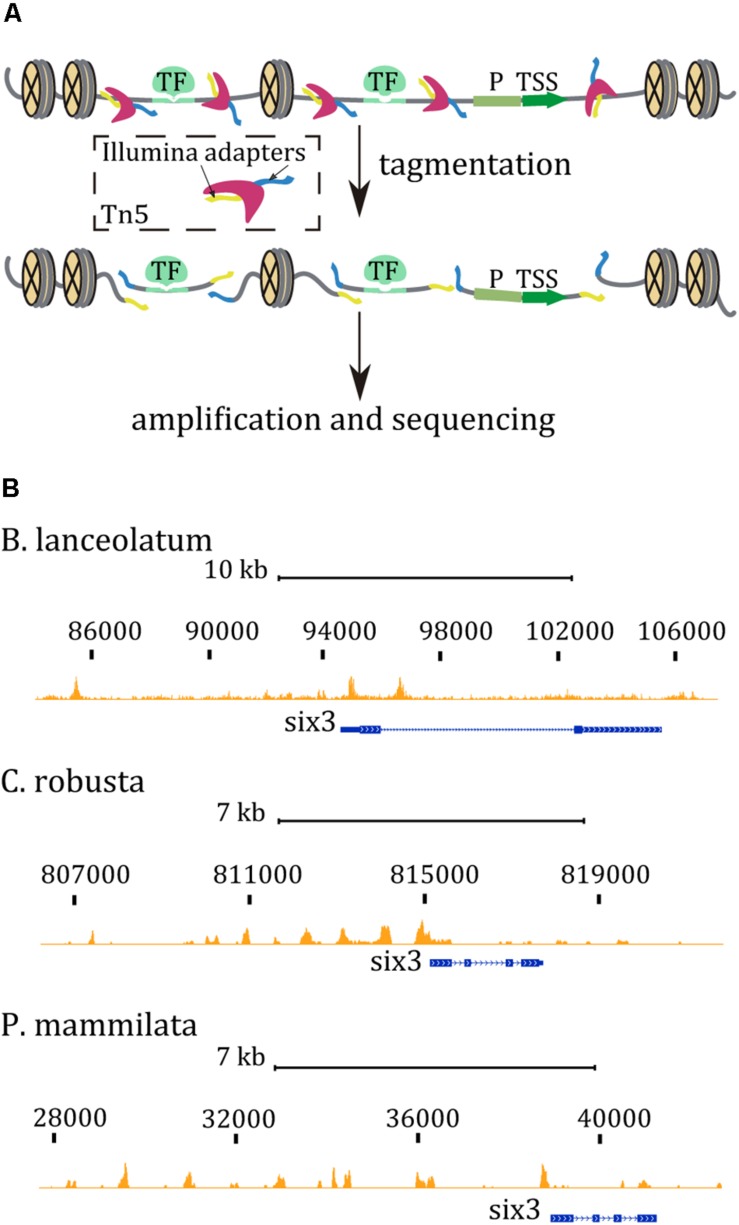
**(A)** ATAC-seq cartoon reaction. The Tn5 transposase (dark pink) inserts two sequencing adapters (yellow and blue) only in accessible regions, between nucleosomes and binding sites for proteins (green), such as transcription factors (TF). Promoter regions (P) and transcription start sites (TSS) are also considered open chromatin regions. Tn5 generates sequencing fragments that can be amplified by PCR. **(B)** ATAC-seq tracks in the *six3* region from chordates embryos at late gastrula stage. ATAC-seq tracks are marked in orange while the gene model is represented in blue. Mapped sequenced reads, <130 bp, identify discrete open chromatin regions mostly upstream the body gene region.

In the last years, the identification of CREs has been boosted by the emergence of next-generation sequencing. Among the new available techniques, we selected the Assay for Transposase-Accessible Chromatin followed by high-throughput sequencing (ATAC-seq; Buenrostro et al., 2013). ATAC-seq is a simple, easy-to-use, and efficient technique for identification of CREs actively bound by TFs in most tissues and organs of different species (Bogdanović et al., 2016; Sebé-Pedrós et al., 2016; Kittelmann et al., 2018; Marlétaz et al., 2018; Ruiz et al., 2018; Madgwick et al., 2019). The assay is based on the hyperactive Tn5 transposase, which preferentially integrates into open chromatin regions, where it cuts DNA into fragments and simultaneously attaches sequencing Illumina adapters to the fragment ends, facilitating the direct construction of the sequencing library (Figure 1A). Sequencing reads are used to infer regions of increased accessibility such as promoters or enhancers, discriminating them from regions occupied by nucleosomes. Unlike ChIP-seq and DNAse-seq, ATAC-seq enables obtaining, in a 1-day procedure, DNA libraries with genome-wide information from fewer than 10^5^ cells. Thus, this technique permits identification of open chromatin regions, offering a much more precise and realistic picture of how functional CREs are distributed in the genome. Moreover, applying this protocol to embryos at specific embryonic stages or to isolated populations of cells allows efficient exploration of the dynamics of regulatory activity across the different developmental stages and tissues. Finally, comparing orthologous regulatory regions in related species will give us the opportunity to find functionally conserved enhancers that are maintained at equivalent genomic positions, even though their sequences have diverged. Consequently, these studies move us closer to studying how changes at CREs are associated with gene expression differences during development and how they contribute to the evolution of novel morphological traits.

Here, we provide a step-by-step protocol with the aim of unifying species-specific procedures recently published (Marlétaz et al., 2018; Madgwick et al., 2019). We also provide detailed information about the handling of embryos, such as *in vitro* fertilization, and about how to obtain the optimal cell lysates specific for each organism. Starting from the cell lysate, the subsequent protocol steps are common for all the organisms used. Then, we propose an easy-to-follow workflow for basic bioinformatic analysis applicable to biological questions, such as identifying differential ATAC-seq signal across development or in embryos with perturbed signaling pathways. To demonstrate the robustness of our analysis, we used recently published ATAC-seq data from wild-type (wt) *Ciona* embryos at the 112-cell stage and embryos treated with CHIR-99021 (Madgwick et al., 2019), a pharmacological activator of the Wnt/β-catenin pathway (Naujok et al., 2014; Wu et al., 2015). This pathway has an ancestral function during primary body axis formation in both bilaterians and non-bilaterians (Petersen and Reddien, 2009; Imai et al., 2000; Creyghton et al., 2010; Loh et al., 2016). Our novel analysis reveals that the ATAC-seq peaks that are more accessible in the embryos treated with the Wnt agonist are enriched for TFs binding motifs (TFBMs) linked with this pathway. Accordingly, these potential CREs are associated with genes that play critical roles during development.

Our results strongly suggest that combining ATAC-seq with embryo perturbation experiments is a powerful method for identification of biological significant CREs critical for embryo development in multiple animal models.

## Materials and Equipment

### Reagents

• 4′-6-Diamidino-2-phenylindole dilactate, DAPI (Invitrogen Thermo Fisher Scientific, San Diego, CA, United States, catalog no.: D3571)
• 4-(2-Hydroxyethyl)piperazine-1-ethanesulfonic acid, HEPES (Sigma-Aldrich Merck, catalog no.: H3375)
• Gentamycin (Gibco Thermo Fisher Scientific, San Diego, CA, United States, catalog no.: 15750060)
• IGEPAL CA-630 (Sigma-Aldrich Merck, catalog no.: I8896)
• Low-melting-point agarose
• MinElute PCR purification kit (Qiagen, catalog no.: 28004)
• Nebnext High-Fidelity 2× PCR master mix (New England Biolabs, Ipswich, MA, United States, catalog no.: M0541S)
• Nextera DNA library Prep kit (Illumina, Cambridge, United Kingdom, catalog no.: FC-121-1030)
• *N*,*N*-Dimethylformamide, C_3_H_7_NO (Sigma-Aldrich Merck, catalog no.: D4551)
• Primers for library amplification. Complete list of primers is available in Supplementary Table 1 of Buenrostro et al. (2013).
• Qubit^TM^ dsDNA BR assay kit (Invitrogen Thermo Fisher Scientific, San Diego, CA, United States, catalog no.: Q32850)
• SYBR^TM^ Green I Nucleic Acid Gel Stain 10,000× concentrate in DMSO (Invitrogen Thermo Fisher Scientific, San Diego, CA, United States, catalog no.: S7563)
• Trypsin from porcine pancreas lyophilized powder (Sigma-Aldrich, catalog no.: T4799).

### Materials

• 0.45-μm bottle top filters with PES membrane (Nalgene Thermo Fisher Scientific, San Diego, CA, United States, catalog no.: 295-4545)
• Cell counting chamber
• 0.2 μm cellulose acetate syringe filter (Sigma-Aldrich Merck, catalog no.: CLS431219)
• Filter tips
• 200-μl PCR tubes
• Petri dish 90 × 14 mm
• 1.5-ml polypropylene DNA low binding conical microcentrifuge tubes
• qPCR consumables, products are specific to the instrument
• Soda-lime glass disposable Pasteur pipettes (VWR, catalog no.: 612-1701)
• Rapid-Flow^TM^ Sterile Disposable Filter Units with 0.2-μm PES Membrane (Nalgene Thermo Fisher Scientific, San Diego, CA, United States, catalog no.: 595-4520)
• 500-μl thin-walled PCR tubes (Invitrogen Thermo Fisher Scientific, San Diego, CA, United States, catalog no.: Q32856).

### Animal Samples

#### Branchiostoma lanceolatum

*Branchiostoma lanceolatum* adults were collected during their breading season in the coast of Argelès-sur-mer, France, according to the previously described method (Fuentes et al., 2004).

#### *Ciona intestinalis* and *Phallusia mammillata*

Adult animals of *Ciona intestinalis* (previously *C. intestinalis* type B; Caputi et al., 2007) and *Phallusia mammillata* were collected from the wild by the marine service provided by Centre de Ressources Biologiques Marines in Roscoff, France.

### Solutions

Attention: all solutions described are prepared with milli-Q quality water, unless otherwise specified.

• NaOH 1 M.
• HEPES 0.5 M pH 8 (500 ml): Dissolve 59.58 g HEPES in 400 ml of water and adjust to pH 8 by adding drops of 1 M NaOH. Complete the volume up to 500 ml with water.
• Artificial sea water-HEPES (ASWH): NaCl 500 mM, Kcl 9 mM, CaCl_2_ 10 mM, MgCl_2_ 24.5 mM, MgSO_4_ 24.5 mM, NaHCO_3_ 2.15 mM, HEPES 5 mM, pH 8.
  To prepare 1 L of ASWH, dissolve 29.22 g NaCl, 0.67 g KCl, 1.11 g CaCl_2_, 2.33 g MgCl_2_, 2.95 g MgSO_4_, and 0.18 g NaHCO_3_ in 1 L of water. Next, add 1 ml of HEPES. Sterilize through 0.2-μm PES membrane and add 0.05 g/L of gentamycin.
    Comments: ASWH may be kept at 18°C to use it with ascidian embryos.
• Filtered seawater: filter natural seawater through bottle top filter with 0.45-μm PES membrane.
  Comments: filtered seawater may be kept at 19°C to use it with *B. lanceolatum* embryos.
• 1% Agarose in ASWH
• 0.8% Agarose in filtered seawater
• DAPI 1000×.
  Comments: 1-ml aliquots can be long term stored at −20°C. The stock solution must be diluted 1:100 with distillated water to reach 10×.
• 20% IGEPAL CA-630 (1 ml): dissolve 200 μl OF IGEPAL CA-630 in 800 μl of water.
  Tips: IGEPAL CA-630 is a detergent difficult to dissolve. Use a platform rocker or vertical rotator to help to dissolve the detergent. Some hours are needed to obtain a homogeneous solution.
    Comments: 20% IGEPAL CA-630 is stored for 1 week at room temperature.
• MgCl_2_ 1 M
• NaCl 4 M
• Tris–HCl 2 M, pH 7.5
• Lysis buffer: 10 mM Tris–HCl, pH 7.4, 10 mM NaCl, 3 mM MgCl_2_, and 0.1% IGEPAL CA-630
  To prepare 1 ml of lysis buffer, mix 5 μl of Tris–HCl 2 M, pH 7.5, 2.5 μl NaCl 4 M, 3 μl MgCl_2_ 1 M, 5 μl 20% IGEPAL CA-630, and 984.5 μl of water.
    Tips: the lysis buffer must be prepared fresh and kept on ice while using it.
• Sodium acetate 3 M, pH 5.3.
• 2× Tagmentation buffer: as alterative to the TD buffer provided by the Nextera kit, a tagmentation buffer can be prepared as follows: 20 mM Tris(hydroxymethyl)aminomethane; 10 mM MgCl_2_; 20% (vol/vol) dimethylformamide.
  To prepare 10 ml of tagmentation buffer, mix 100 μl of Tris–HCl 2 M pH 7.5, 100 μl MgCl_2_ 1 M, and 6 ml of water. Finally, add 2 ml *N*,*N*-dimethylformamide and increase the volume up to 10 ml with water. Sterilize the solution by filtration using a 0.2-μm cellulose acetate syringe filter.
    Tips: TAPS-NaOH 2 M, pH 8.5, can be used as an alternative to Tris–HCl to prepare the buffer.
    Comments: 2× tagmentation buffer may be kept at −20°C no longer than 6 months.
• 0.1% Trypsin in ASWH.
  Comments: this solution may be kept at −20°C for long-term storage.

### Equipment

• Real-Time PCR Detection System
• Incubator with accurate temperature settings in an interval from 5 to 60°C
• Qubit^TM^ 3.0 Fluorometer (Invitrogen Thermo Fisher Scientific, San Diego, CA, United States)
• Refrigerated centrifuge
• Thermal cycler
• Compound fluorescence microscope with UV filter.

### Software and Bioinformatic Tools

• Bedtools version 2.26.0^1^
• Bowtie2 version 2.2.6^2^
• DESeq2 version 1.18.1^3^
• Homer version 4.9.1^4^
• IDR version 2.0.3^5^
• MACS2 version 2.1.1.20160309^6^
• PHANTER^7^
• R version 3.4.2 “Short summer”^8^
• Samtools version 1.3^9^.

### Genome and Annotation

#### B. lanceolatum

Genome assembly was generated and described in Marlétaz et al. (2018). UCSC hub and annotation files are accessible at http://amphiencode.github.io/ (Table 1).

**TABLE 1:** Genomes.

**Genome size (Mb)**		
*B. lanceolatum*	BraLan2	495
*C. robusta*	Cirobu_KH	115
*P. mammillata*	MTP2014	234

#### *C. intestinalis* and *P. mammillata*

*C. intestinalis* and *P. mammillata* genome are available for users in http://www.aniseed.cnrs.fr/ (Table 1). For the analysis described in the results section, Apr. 2011; Kyoto KH/ci3 *Ciona robusta* was used. Genome and the transcription start site (TSS) information were downloaded from UCSC Genome Browser^10^.

### Procedure

#### *In vitro* Fertilization

##### *B. lanceolatum* samples

To obtain *B. lanceolatum* eggs and fertilize them, follow the protocol previously described (Fuentes et al., 2004, 2007). Around 30 min after fertilization, collect the embryos in Petri dishes coated with a thin layer of 0.8% agarose and filled with filtered seawater. Dechorionate embryos manually by pipetting them toward the border of the dish. Incubate embryos at 19°C. Check the development of the embryos 3 h after fertilization. Gently transfer healthy embryos to a new Petri dish with filtered seawater using tips with cut ends, previously incubated for several hours in filtered seawater.

##### *C. intestinalis* and *P. mammillata* samples

To obtain ascidian embryos, incubate eggs from both species with activated sperm for 8 min. *Phallusia* eggs needed to be dechorionated prior fertilization by incubation in 0.1% trypsin in ASWH, while *Ciona* eggs were dechorionated after fertilization (Christiaen et al., 2009). Keep developing embryos at 18°C in ASWH in a Petri dish coated with a thin layer of 1% agarose melted in ASWH.

#### Collection of Embryos

We recommend starting ATAC-seq experiments with 100,000–180,000 cells for amphioxus and tunicate species. Use half of the cells (50,000–90,000 cells) for the actual tagmentation step and use the rest of the cells to estimate the number of nuclei in each sample. Optimizing the number of cells to use for the transposition is crucial, since it strongly affects library quality. In fact, using too few cells per experiment leads to over-transposition, while using too many cells produces incomplete transposition and over-sized DNA fragments.

##### *B. lanceolatum* samples

Keep embryos at 19°C until they reach the desired developmental stage. Take the appropriate number of embryos according to their developmental stage (Table 2) and transfer them to a 1.5 ml microtube.

**TABLE 2:** Number of *B. lanceolatum* embryos, according to developmental stages, to achieve recommended cell number (100,000–180,000 cells) for ATAC-seq.

**Developmental stage (hpf)**	**Number of embryos**
8	200
15	50
36	25
60	25

##### *C. instestinalis* and *P. mammillata* samples

(i) Keep *Ciona* and *Phallusia* embryos at 18°C until they reach the desired stage (Hotta et al., 2007). Collect embryos from the Petri dish and transfer them to a 1.5-ml microtube with 200 μl of ASWH at the bottom. To get a sufficient number of cells, we estimated that the number of dechorionated embryos in a packed volume of 100 μl was in the order of 10,000–20,000. Table 3 shows the volume of embryos collected depending on the developmental stage.
  **TABLE 3:** Volume of collected ascidian embryos for the estimation of the recommended cell number (100,000–180,000 cells) needed for ATAC-seq.

**Developmental stage**	**Volume of embryos (μL)**
16 cells	30–50
32 cells	20–25
64 cells	10–12
112 cells	7–10
Late gastrula	7–10
Mid neurula	7–10

Attention: avoid air bubbles while pipetting since exposure of embryos to air dismantles embryonic structures.

(ii) Swirl the dish to group embryos, collect them using a glass Pasteur pipette (see Note 1), and transfer them to the 1.5-ml microtube with ASWH.

Attention: from here, keep samples on ice, and use gloves and filter tips.

#### Cell Lysis

##### *B. lanceolatum* samples

(i) Centrifuge the sample at maximum speed at 4°C for 1 min and carefully discard supernatant.
(ii) Add 50 μl of cold lysis buffer and lyse the cells by pipetting up and down with P200 micropipette. For optimal cell lysis, proceed immediately to next step.
(iii) Transfer 8 μl of lysate in three new 1.5-ml microtubes and centrifuge them at 500 × *g* at 4°C for 10 min. During this 10 min, use the remaining 26 μl to count the cells for transposition: add 2.6 μl of 10× DAPI to 26 μl of the remaining sample and place 10–12 μl of the mix in a cell counting chamber and count cells in a compound fluorescence microscope using UV light.
(iv) Remove supernatant carefully. Use the amount of sample needed to reach the optimal number of cells for the assay and continue with tagmentation step.

##### *C. instestinalis* and *P. mammillata* samples

Immediately place the microtube in a cold centrifuge and spin it at 500 × *g* and 4°C for 5 min to pellet the embryos. Remove the supernatant carefully.

(i) Wash twice with 200 μl of ASWH by spinning down the cells at 500 × *g* and 4°C for 5 min.
(ii) Resuspend the pellet in 100 μl of cold lysis buffer and pipet up and down with a P200 micropipette. The lysate should be clear. Lysing *Ciona* and *Phallusia* embryos by pipetting can be difficult at early stages like 16-cell and 32-cell stages due to the large amount of yolk per cell. This may take around 3 min of vigorous pipetting with a P200 micropipette. For optimal cell lysis, incubate the cells no longer than indicated and proceed immediately to the next step.
(iii) Transfer 50, 20, and 10 μl of the lysate into new 1.5-ml microtubes and centrifuge them at 500 × *g* at 4°C for 10 min. Meanwhile, use the remaining 20 μl to count the cells to estimate the number of cells to use in the transposition reaction: add 2 μl of 10× DAPI to 20 μl of the remaining sample. Load the mix in a cell counting chamber and count cells in a compound fluorescence microscope using UV light.
(iv) Remove supernatant carefully with a micropipette. Use the aliquots required (50, 20, and 10 μl) to reach 50,000–90,000 nuclei required in the tagmentation step.

Attention: at this step, the nuclear pellet is hard to see. Avoid touching the bottom of the microtube, where the nuclei are pelleted, to not remove them.

The following procedures can be applied to all samples, regardless of species.

#### Tagmentation

(1) Keep samples on ice. Prepare tagmentation reaction mix (50 μl) by combining:
  25 μl 2× TD tagment DNA buffer
  2.5 μl TDE1 tagment DNA enzyme
  22.5 μl autoclaved milli-Q water
(2) Gently resuspend the nuclear pellet in the tagmentation reaction mix by pipetting. If more than one aliquot is used to achieve the required cell number, resuspend aliquots by using the same 50-μl tagmentation reaction mix.
(3) Incubate the tagmentation reaction at 37°C for 30 min.
(4) Purify fragmented DNA by using the Qiagen MinElute PCR purification kit, following the manufacturer’s instructions. Firstly, add 2.5 μl of 3 M sodium acetate pH 5.3 to the sample (see Note 2) prior to purification, then continue with the instructions. Elute tagmented DNA in 20 μl of Elution Buffer (see Note 3).

Comment: purified DNA fragments can be stored at −20°C or immediately subjected to the library preparation step (Steps 5 or 6).

#### Library Preparation

(5) (*Optional step*) Determine the optimal PCR cycle number for the final ATAC library preparation. The optimal number of cycles is the Ct number obtained performing a qPCR plus one cycle more. Ct number is increased one cycle more to scale reaction and keep ATAC library preparation in exponential phase (see Notes 4, 5).
(I) To do that, prepare the following PCR mix (10 μl):
  1 μl tagmented DNA
  1 μl forward primer 5 μM (see Note 5)
  1 μl reverse primer 5 μM (see Note 5)
  1 μl SYBR Green I Nucleic Acid Gel Stain 10×
  5 μl NEBNext High-Fidelity 2× PCR Master Mix
  1 μl autoclaved milli-Q water
(II) Perform qPCR with the following thermocycling conditions:
  (1) 72°C, 5 min
  (2) 98°C, 30 s
  (3) 98°C, 10 s
  (4) 63°C, 30 s
  (5) 72°C, 1 min
  (6) Repeat steps 3–5, 25 times
  (7) Hold at 4°C
(6) To generate the ATAC library, prepare the following PCR mix (50 μl):
  19 μl tagmented DNA
  1 μl autoclaved milli-Q water
  2.5 μl forward primer 25 μM (see Note 5)
  2.5 μl reverse primer 25 μM (see Note 5)
  25 μl NEBNext High-Fidelity 2× PCR Master Mix.

Comment: if Step 5 was omitted, use all 20 μl of eluted DNA from Step 6 and do not add autoclaved milli-Q water in the PCR reaction mix.

(7) Amplify DNA fragments with the following thermocycling conditions:
  (1) 72°C, 5 min
  (2) 98°C, 30 s
  (3) 98°C, 10 s
  (4) 63°C, 30 s
  (5) 72°C, 1 min
  (6) Repeat Steps 3–5, estimated number of cycles
  (7) Hold at 4°C
(8) Purify amplified library using Qiagen MinElute PCR purification kit, following the manufacturer’s instructions. Firstly, add 2.5 μl of 3 M sodium acetate pH 5.3 to each sample (see Note 2) and then continue with the instructions. Elute the purified DNA library in 20 μl Elution Buffer (see Note 3).

Comment: purified DNA can be stored at −20°C.

#### Quality Controls

(9) Use 1 μl of amplified DNA library to measure DNA sample concentration using Qubit dsDNA BR Assay kit with 500-μl thin-walled tubes. Follow manufacturer’s instructions (see Note 6).
(10) Run 2–5 μl of the amplified DNA library on 2% agarose gel to check tagmentation. A smear covering 100 bp to 1 kb should be observed. Depending on the quality of the library, besides nucleosome-free fragments (<200 bp), a mono- (200 bp) and di-nucleosome (400 bp) band pattern may be observed.

#### DNA Library Sequencing

(11) The required sequencing depth for the generation of a genome-wide profile with good footprinting signal depends on the genome size of the organism under study. In our experiments, we have used between 40 and 100 million paired-end reads per DNA library.

### Data Analysis

The data used was obtained from *B. lanceolatum* (GSE106428), *C. intestinalis* (PRJNA474983), and *P. mammillata* datasets (PRJNA474750). After sequencing, align paired-end reads without adapter sequences against the reference genome using Bowtie2 (Langmead and Salzberg, 2012) with *-X 2000 –no-mixed — no-unal* parameters. This procedure allows the retention of reads that are separated <2 kb. Then, remove PCR artifacts and duplicates using the tool *rmdup*, available in the Samtools toolkit (Li et al., 2009). In order to detect the position where transposase binds to the DNA, corresponding to accessible chromatin, read start sites need to be offset by +4 or by -5 bp in the plus or minus strand, respectively. Select read pairs that have an insert <130 bp, since they correspond to nucleosome-free reads. Next, generate BigWig files using genomecov from Bedtools (Quinlan and Hall, 2010) and wigtoBigWig tool from UCSC. These files can be uploaded to a genome browser, in order to explore the data. Call peaks using filtered read files using the MACS2 tool (Zhang et al., 2008). The parameters of this peak calling are *–nomodel –shift -45 –extsize 100* and the genome size of the correspondent organism. For each of these peak callings, select the first 500,000 peaks ranked by *p*-value. Use the irreproducible discovery rate (IDR) framework (Li et al., 2011) for the identification of high confidence peaks based on replicate information, with the following parameters: *–input-file-type narrowpeak –rank p.value –soft-idr-threshold 0.1* and the peak calling coming from MACS2. Select high confidence peaks with and IDR global value of 0.01 for further analysis. The proportion of reads inside peaks can be calculated with intersectBed from Bedtools (Quinlan and Hall, 2010), using the high confidence set of peaks as reference and the filtered reads as query, using the parameter *-c*.

Use the R package DESeq2 (Love et al., 2014) to assess differences between the accessibility of peaks between wt and a test condition. For each comparison, merge all the high confidence peaks of each condition that is going to be analyzed. Then, compute the number of reads inside the peaks for each experimental replicate. Select those peaks that show a *p-*value under 0.05 as differentially accessible peaks. Then, differentially accessible peaks can be associated with their putative target genes using “Genomic Regions Enrichment of Annotations Tool” (GREAT; McLean et al., 2010) or the closestBED tool (Quinlan and Hall, 2010) utility from BEDtools. For the analysis described in the section “Results,” we used the latter one with the parameters *-D ref -iu -non-amecheck -k 1*. The consecutive gene ontology (GO) analysis can be performed with PANTHER (Mi et al., 2019). Finally, to find TFBM enrichment in ATAC-seq peaks use the script FindMotifsGenome.pl from HOMER software (Heinz et al., 2010), selecting the set of desired TFBMs with the parameter *-mset*. As background model, merge high confidence peaks from all ATAC-seq datasets.

## Anticipated Results

ATAC-seq assays have been performed in wt embryos of amphioxus and tunicates at different developmental stages mentioned in Tables 2, 3, respectively. ATAC-seq data can be easily visualized in a genome browser. For instance, ATAC-seq tracks generated in amphioxus and the equivalent stage in tunicate embryos are shown in Figure 1B. Interestingly, the ATAC-seq signal in the *six3* region identified discrete regions in all three cases. There are more prominent accessible chromatin regions upstream of the *six3* gene rather than downstream. In all cases, an ATAC signal is overlapping the TSS or promoter region of the *six3* gene. The number of peaks per experiment and the percentage of mapped reads in these peaks are shown in Table 4. In *B. lanceolatum* samples, the number of peaks was between 16,000 and 48,000, while in both tunicate species, it was between 3,000 and 10,000. The percentage of nucleosome-free reads found in peaks was quite variable in all samples of the three species, but similar between most replicates.

**TABLE 4:** Number of conservative peaks obtained by IDR framework and percentage of nucleosome-free reads in conservative peaks.

***B. lanceolatum***								
wt samples	8 hpf		15 hpf		36 hpf		60 hpf	
Number of peaks	16,697		33,921		32,477		48,635	
% reads in peaks	rep1	rep2	rep1	rep2	rep1	rep2	rep1	rep2
	10.54	14.45	29.38	44.24	26.32	29.31	50.66	49.12

***C. intestinalis***								

wt samples	64 cells		112 cells		Late gastrula			
Number of peaks	6621		3882		10,652			
% reads in peaks	rep1	rep2	rep1	rep2	rep1	rep2		
	34.43	35.77	33.57	34.79	53.80	45.75		

***P. mammillata***								

308 wt samples	64 cells		112 cells		Late gastrula		Mid neurula	
Number of peaks	3594		3202		5985		6203	
% reads in peaks	rep1	rep2	rep1	rep2	rep1	rep2	rep1	rep2
	28.37	20.33	18.19	20.68	29.43	30.91	36.35	28.50

We also provide an example of using ATAC-seq for the identification of CREs controlled by the Wnt signaling pathway. For that, we used recently generated ATAC-seq libraries from *Ciona* embryos at the 112-cell stage as a control and embryos treated with 4 μM CHIR-99021, a GSK3 inhibitor acting as an activator of the canonical Wnt pathway (Madgwick et al., 2019). Here, we showed further bioinformatic analysis that allows the identification of differential ATAC-seq peaks between control and treated embryos, the TFBMs enrichment in those peaks, and the GO of the associated genes.

**FIGURE 2:**
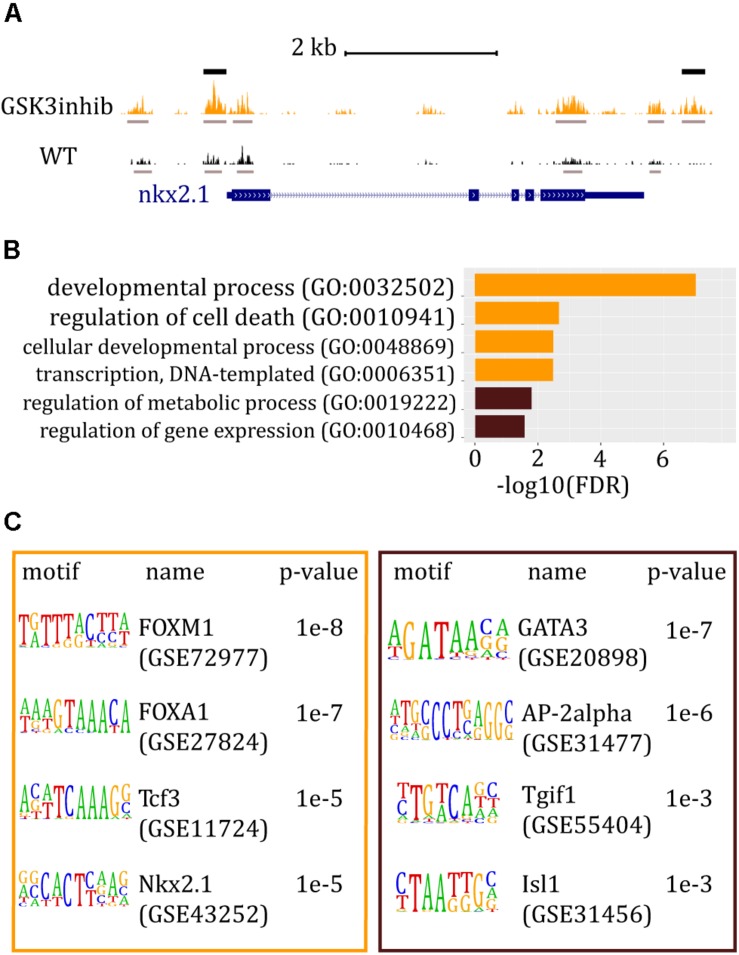
ATAC-seq data and analysis of *Ciona* embryos at the 112-cell stage after Wnt/β-catenin signaling pathway was altered. **(A)** Snapshot from the UCSC Browser of *Ciona* genome showing the ATAC-seq signal around the *nkx2.1* gene. The gene model is represented in blue. ATAC-seq track of embryos treated with a GSK3 inhibitor is shown in orange whereas ATAC-seq track of wt embryos is in black. Gray bars mark called peaks, whereas black bars indicate peaks that are significantly higher in treated embryos. **(B)** Most relevant GO results generated by PANTHER. In orange: GO term bars related to genes associated with chromatin regions significantly more accessible in embryos where the activity of Wnt pathway was increased. In brown: GO term bars related to genes associated with more accessible chromatin regions in wt embryos. **(C)** HOMER results for peaks differentially represented between wt and the perturbed condition. In the orange box: known motif enrichment results of peaks significantly more represented when Wnt/β-catenin signaling pathway was increased. In the brown box: known motif enrichment results of peaks significantly more represented in wt embryos.

By genome-wide searching for peaks that are differentially accessible between untreated and treated embryos upon Wnt activation, we find 57 more accessible and 40 less accessible ATAC-seq peaks. In fact, we identified two discrete regions in the *nkx2.1* locus, which were significantly affected by the treatment (Figure 2A). GO analysis of the genes associated with more accessible chromatin regions underlined the role of Wnt activation in embryo development, as expected. In contrast, genes associated with less accessible ATAC-seq peaks were mostly related to metabolic processes (Figure 2B). Our GO analysis was consistent with differentially expressed gene analysis obtained with another GSK3 inhibitor, known as BIO (Wu et al., 2015). Enrichment analysis, used to predict potential TFBMs in differentially accessible ATAC-seq peaks, showed that Fox, Tcf, and Nkx TFBMs were the most represented in the set of more accessible ATAC-seq peaks (Figure 2C). The enrichment of Tcf, which is an effector TF of the canonical Wnt signaling pathway, validating that the treatment indeed promoted this pathway. On the contrary, Gata motifs were found enriched in the set of less accessible peaks (Figure 2C), confirming the previously reported antagonism between Gata and Wnt in *Ciona* (Imai et al., 2016).

## Conclusion

Here, we present a comprehensive ATAC-seq protocol that is highly efficient for the identification of *cis*-regulatory regions operating during embryogenesis in several marine species. The main advantages of ATAC-seq are the short experimental time and the low amount of starting material needed. We show that this protocol nicely works in embryos from three species, *B. lanceolatum*, *C. intestinalis*, and *P. mammillata*. Moreover, protocols for embryo collection and for experimental procedure are described in detail. Our results pointed out the robustness of the experimental procedure in marine invertebrate embryos and the great utility of the ATAC-seq technique. Since we have succeeded in generating data from the different species tested at diverse developmental stages, we consider this protocol applicable to other species and/or stages. Indeed, in our laboratory, we have successfully applied this method on two different echinoderms and one hemichordate species. Additionally, we present some bioinformatic tools that can be used to analyze and visualize ATAC-seq data (Figure 1B) and, more importantly, identify regulatory regions that respond to alterations in signaling pathways (Figure 2).

Our work demonstrates that performing ATAC-seq in embryos treated with drugs that perturb signaling pathways is a powerful strategy for the genome-wide identification of CREs mediating the activity of different signaling pathways. Indeed, our laboratory is currently successfully applying this approach to study multiple signaling pathways in several invertebrate and vertebrate species. Therefore, one of the major goals of this protocol is to facilitate and encourage the community to use this approach to study their favorite systems and pathways.

### Notes

(1) Glass Pasteur pipettes are previously dipped in milli-Q water overnight in order to avoid embryos sticking to the glass.
(2) 3 M sodium acetate, pH 5.3, decreases the pH of the samples. This helps to recover a high quantity of DNA fragments during the purification step, according to the MinElute PCR purification kit manufacturer’s instructions. It is important to not add pH indicator in the binding buffer, because it interferes with downstream sequencing.
(3) Warming the Elution Buffer at 37°C before use is recommended.
(4) To obtain good quality ATAC-seq libraries in this work, we determined that 13–15 PCR cycles were optimal. However, to avoid experimental variations and PCR artifacts, it is recommended to determine the optimal PCR cycle number in every experiment independently.
(5) A complete list of PCR primers complementary to Nextera adaptors is available in Supplementary Table 1 of Buenrostro et al. (2013). In order to multiplex different samples in the same sequencing lane, variable barcoded reverse primers need to be used.
(6) A minimum concentration of 20 ng/μl is recommended for next-generation sequencing.

## DATA AVAILABILITY STATEMENT

All datasets generated for this study are included in the article/supplementary material.

## Author Contributions

SJ-G, SB, HE, and JG-S developed the protocol for *B. lanceolatum* samples. MM, PL, and JG-S designed the protocol for *C. intestinalis* and *P. mammillata*, and conceived and designed experiments in *C. intestinalis* embryos. MM and AM performed the ATAC-seq assays in *Ciona* and *Phallusia* embryos. SJ-G and MM executed the bioinformatic analysis of *B. lanceolatum*, *C. intestinalis*, *and P. mammillata* ATAC-seq data. MM performed the computational analysis of differential ATAC-seq data of *Ciona* and data interpretation, and wrote the draft of the manuscript. SJ-G wrote the sections related to the ATAC-seq assays in *B. lanceolatum* samples. All authors contributed to the manuscript revision.

## Conflict of Interest

The authors declare that the research was conducted in the absence of any commercial or financial relationships that could be construed as a potential conflict of interest.

## Footnotes

1 https://github.com/arq5x/bedtools2/releases/

2 http://bowtie-bio.sourceforge.net/bowtie2/index.shtml

3 https://bioconductor.org/packages/release/bioc/html/DESeq2.html

4 http://homer.ucsd.edu/homer/motif/

5 https://github.com/nboley/idr

6 https://pypi.org/project/MACS2/

7 http://www.pantherdb.org/

8 https://www.r-project.org/

9 http://www.htslib.org/

10 https://genome-euro.ucsc.edu/cgi-bin/hgTables?hgsid=231102165_vTWE7h7DGoTv0QlthOAGgKbTLi14

## References

[B1] Bogdanović O. Smits A. H. de la Calle-Mustienes E. Tena J. J. Ford E. Williams R. (2016). Active DNA demethylation at enhancers during the vertebrate phylotypic period. *Nat. Genet.* 48 417–426. 10.1038/ng.3522 26928226 PMC5912259

[B2] Buenrostro J. D. Giresi P. G. Zaba L. C. Chang H. Y. Greenleaf W. J. (2013). Transposition of native chromatin for fast and sensitive epigenomic profiling of open chromatin, DNA-binding proteins and nucleosome position. *Nat. Methods* 10 1213–1218. 10.1038/nmeth.2688 24097267 PMC3959825

[B3] Caputi L. Andreakis N. Mastrototaro F. Cirino P. Vassillo M. Sordino P. (2007). Cryptic speciation in a model invertebrate chordate. *Proc. Natl. Acad. Sci. U.S.A.* 104 9364–9369. 10.1073/pnas.0610158104 17517633 PMC1890500

[B4] Christiaen L. Wagner E. Shi W. Levine M. (2009). Isolation of sea squirt (Ciona) gametes, fertilization, dechorionation, and development. *Cold Spring Harb. Protoc.* 4 1–7. 10.1101/pdb.prot5344 20150091

[B5] Creyghton M. P. Cheng A. W. Welstead G. G. Kooistra T. Carey B. W. Steine E. J. (2010). Histone H3K27ac separates active from poised enhancers and predicts developmental state. *Proc. Natl. Acad. Sci. U.S.A.* 107 21931–21936. 10.1073/pnas.1016071107 21106759 PMC3003124

[B6] Fuentes M. Benito E. Bertrand S. Paris M. Mignardot A. Godoy L. (2007). Insights into spawning behavior and development of the European amphioxus (Branchiostoma Lanceolatum). *J. Exp. Zool. Part B* 308 484–493. 10.1002/jez.b.21179 17520703

[B7] Fuentes M. Schubert M. Dalfo D. Candiani S. Benito E. Gardenyes J. (2004). Preliminary observations on the spawning conditions of the European Amphioxus (Branchiostoma Lanceolatum) in captivity. *J. Exp. Zool. Part B* 302 384–391. 10.1002/jez.b.20025 15287102

[B8] Heinz S. Benner C. Spann N. Bertolino E. Lin Y. C. Laslo P. (2010). Simple combinations of lineage-determining transcription factors prime cis-regulatory elements required for macrophage and B cell identities. *Mol. Cell* 38 576–589. 10.1016/j.molcel.2010.05.004 20513432 PMC2898526

[B9] Hotta K. Mitsuhara K. Takahashi H. Inaba K. Oka K. Gojobori T. (2007). A web-based interactive developmental table for the Ascidian Ciona intestinalis, including 3D real-image embryo reconstructions: I. from fertilized egg to hatching Larva. *Dev. Dyn.* 236 1790–1805. 10.1002/dvdy.21188 17557317

[B10] Imai K. Takada N. Satoh N. Satou Y. (2000). (Beta)-catenin mediates the specification of endoderm cells in Ascidian embryos. *Development* 127 3009–3020. 10862739 10.1242/dev.127.14.3009

[B11] Imai K. S. Hudson C. Oda-Ishii I. Yasuo H. Satou Y. (2016). Antagonism between β-Catenin and Gata.a sequentially segregates the germ layers of Ascidian embryos. *Development* 143 4167–4172. 10.1242/dev.141481 27707797

[B12] Kittelmann S. Buffry A. D. Franke F. A. Almudi I. Yoth M. Sabaris G. (2018). Gene regulatory network architecture in different developmental contexts influences the genetic basis of morphological evolution. *PLoS Genet.* 14:e1007375. 10.1371/journal.pgen.1007375 29723190 PMC5953500

[B13] Langmead B. Salzberg S. L. (2012). Fast gapped-read alignment with Bowtie 2. *Nature Methods* 9 357–359. 10.1038/nmeth.1923 22388286 PMC3322381

[B14] Li H. Handsaker B. Wysoker A. Fennell T. Ruan J. Homer N. (2009). The sequence alignment/Map format and SAMtools. *Bioinformatics* 25 2078–2079. 10.1093/bioinformatics/btp352 19505943 PMC2723002

[B15] Li Q. Brown J. B. Huang H. Bickel P. J. (2011). Measuring reproducibility of high-throughput experiments. *Ann. Appl. Stat.* 5 1752–1779. 10.1214/11-AOAS466

[B16] Loh K. M. Van Amerongen R. Nusse R. (2016). Generating cellular diversity and spatial form?: wnt signaling and the evolution of multicellular animals. *Dev. Cell* 38 643–655. 10.1016/j.devcel.2016.08.011 27676437

[B17] Long H. K. Prescott S. L. Wysocka J. (2016). Ever-changing landscapes: transcriptional enhancers in development and evolution. *Cell* 167 1170–1187. 10.1016/j.cell.2016.09.018 27863239 PMC5123704

[B18] Love M. I. Huber W. Anders S. (2014). Moderated estimation of fold change and dispersion for RNA-Seq data with DESeq2. *Genome Biol.* 15 1–21. 10.1186/s13059-014-0550-8 25516281 PMC4302049

[B19] Madgwick A. Magri M. S. Dantec C. Gailly D. Fiuza U. M. Guignard L. (2019). Evolution of embryonic cis-regulatory landscapes between divergent Phallusia and Ciona ascidians. *Dev. Biol.* 448 71–87. 10.1016/j.ydbio.2019.01.003 30661644

[B20] Marlétaz F. Firbas P. N. Maeso I. Tena J. J. Bogdanovic O. Perry M. (2018). Amphioxus functional genomics and the origins of vertebrate gene regulation. *Nature* 564 64–70. 10.1038/s41586-018-0734-6 30464347 PMC6292497

[B21] McLean C. Y. Bristor D. Hiller M. Clarke S. L. Schaar B. T. Lowe C. B. (2010). GREAT improves functional interpretation of Cis-regulatory regions. *Nat. Biotechnol.* 28 495–501. 10.1038/nbt.1630 20436461 PMC4840234

[B22] Mi H. Muruganujan A. Ebert D. Huang X. Thomas P. D. (2019). PANTHER Version 14: more Genomes, a new PANTHER GO-slim and improvements in enrichment analysis tools. *Nucleic Acids Res.* 47 D419–D426. 10.1093/nar/gky1038 30407594 PMC6323939

[B23] Naujok O. Lentes J. Diekmann U. Davenport C. Lenzen S. (2014). Cytotoxicity and activation of the wnt/beta-catenin pathway in mouse embryonic stem cells treated with four GSK3 inhibitors. *BMC Res.* 7:273. 10.1186/1756-0500-7-273 24779365 PMC4008422

[B24] Petersen C. P. Reddien P. W. (2009). Wnt signaling and the polarity of the primary body axis. *Cell* 139 1056–1068. 10.1016/j.cell.2009.11.035 20005801

[B25] Quinlan A. R. Hall I. M. (2010). BEDTools: a flexible suite of utilities for comparing genomic features. *Bioinformatics* 26 841–842. 10.1093/bioinformatics/btq033 20110278 PMC2832824

[B26] Ruiz J. L. Tena J. J. Bancells C. Cortés A. Gómez-Skarmeta J. L. Gomez-Díaz E. (2018). Characterization of the accessible genome in the human malaria parasite *Plasmodium falciparum*. *Nucleic Acids Res.* 46 9414–9431. 10.1093/nar/gky643 30016465 PMC6182165

[B27] Sebé-Pedrós A. Ballaré C. Parra-Acero H. Chiva C. Tena J. J. Sabidó E. (2016). The dynamic regulatory genome of capsaspora and the origin of animal multicellularity. *Cell* 165 1224–1237. 10.1016/j.cell.2016.03.034 27114036 PMC4877666

[B28] Spitz F. Furlong E. M. (2012). Transcription factors: from enhancer binding to developmental control. *Nat. Rev. Genet.* 13 613–626. 10.1038/nrg3207 22868264

[B29] Wu Y. Liu F. Liu Y. Liu X. Ai Z. Guo Z. (2015). GSK3 inhibitors CHIR99021 and 6-Bromoindirubin-3’-Oxime inhibit MicroRNA maturation in mouse embryonic stem cells. *Sci. Rep.* 5 22–24. 10.1038/srep08666 25727520 PMC4345320

[B30] Zhang Y. Liu T. Meyer C. A. Eeckhoute J. Johnson D. S. Bernstein B. E. (2008). Model-Based analysis of ChIP-Seq (MACS). *Genome Biol.* 9:R137. 10.1186/gb-2008-9-9-r137 18798982 PMC2592715

